# A retrospective study of accuracy and usefulness of electrophysiological exercise tests

**DOI:** 10.1007/s00415-023-12110-5

**Published:** 2023-12-06

**Authors:** Vesa Periviita, Manu Jokela, Johanna Palmio, Bjarne Udd

**Affiliations:** 1 Department of Clinical Neurophysiology, Tampere University Hospital, Tampere, Finland; 2 Neuromuscular Research Center, Tampere University and University Hospital, Tampere, Finland; 3 Neurocenter, Turku University Hospital, Turku, Finland; 4 Neurology, Clinical Medicine, University of Turku, Turku, Finland; 5 Folkhälsan Research Center, Helsinki, Finland

**Keywords:** Channelopathies, Muscular diseases, Neurophysiology, Observational study

## Abstract

**Objectives:**

This study aimed to determine the usefulness of electrophysiological exercise tests. The significance of slightly abnormal exercise tests was also examined.

**Methods:**

We identified all the patients who had undergone exercise testing between February 2007 to June 2022 in Tampere University Hospital, Finland. Their medical records after diagnostic workup and exercise test reports were reviewed. A binary logistic regression was performed to evaluate the association between positive test result in short exercise test, long exercise test, or short exercise test with cooling and genetically confirmed skeletal muscle channelopathy or myotonic disorder.

**Results:**

We identified 256 patients. 27 patients were diagnosed with nondystrophic myotonia, periodic paralysis, myotonic dystrophy type 1, myotonic dystrophy type 2, or other specified myopathy. 14 patients were suspected to have a skeletal muscle channelopathy, but pathogenic variants could not be identified. The remaining 215 patients were diagnosed with other conditions than skeletal muscle channelopathy or myotonic disorder. The combined sensitivity of exercise tests was 59.3% and specificity 99.1%. Abnormal exercise test result was associated with increased risk of skeletal muscle channelopathy or myotonic disorder (OR 164.3, 95% CI 28.3–954.6, *p* < 0.001).

**Conclusions:**

Electrophysiological exercise test is not optimal to exclude skeletal muscle channelopathy. It may be useful if a skeletal muscle channelopathy is suspected and genetic testing is negative or indeterminate and further evidence is required. Slightly abnormal exercise test results are possible in various conditions and result from different aetiologies. There is a demand for neurophysiological studies with higher sensitivity to detect skeletal muscle channelopathies.

## Introduction

Skeletal muscle channelopathies are rare and heterogeneous disorders. Studies from United Kingdom and Netherlands estimated the prevalence of skeletal muscle channelopathies to be 2:100 000 [1, 2]. Regional prevalence rates of specific skeletal muscle channelopathies may vary considerably [3–5]. Skeletal muscle channelopathies are caused by gene variants encoding sodium channel (*SCN4A*), chloride channel (*CLCN1*), calcium channel (*CACNA1S*), or potassium channels (*KCNJ2* and *KCNJ18*) [6]. These disorders have been traditionally divided into nondystrophic myotonias (NDMs) and periodic paralyses (PPs). NDMs include myotonia congenita (MC), paramyotonia congenita (PC), and sodium channel myotonia (SCM). Periodic paralyses include hyperkalemic periodic paralysis (hyperPP), hypokalemic periodic paralysis (hypoPP), and Andersen-Tawil syndrome [7]. Although rare, other phenotypes have also been associated with these genes. *SCN4A* variants are a well-known cause of PC, SCM, hyperPP, and hypoPP [8], but can also result in congenital myopathy [9] and myasthenia [10–12]. *CACNA1S* variants are known to cause hypokalemic periodic paralysis [13] and susceptibility to malignant hyperthermia [14, 15], but specific variants have also been associated with congenital myopathy [16].

Distinct from the NDMs are the myotonic dystrophies with progressive muscle wasting and multi-organ involvement [13, 17]. Myotonic dystrophies include myotonic dystrophy type 1 (DM1) and myotonic dystrophy type 2 (DM2). DM1 is caused by repeat expansions in *DMPK* and DM2 is caused by repeat expansions in *CNBP*. The core symptoms of DM1 are muscle weakness, myotonia, and cataracts in the most prevalent adult-onset form of the disease. The clinical phenotype is highly variable in DM2 [17], and especially milder cases of DM2 may be confused with NMDs [18, 19]. Global prevalence of DM1 has been estimated to be 9:100 000 and prevalence of DM2 2:100 000 [20]. The estimates vary greatly depending on the studied population [17, 20]. The estimated prevalence in Finland is higher than global estimation, and DM2 is more frequent than DM1 [21].

Electrophysiological exercise tests [22, 23] have been available in Tampere University Hospital since 2007. They may help to distinguish between NDMs [22, 23] and be helpful if genetic testing is negative or indeterminate [24]. The parameter measured has varied in different studies [25]. Fournier and colleagues considered changes in CMAP amplitude between − 10% to + 20% of the pre-exercise value normal in short exercise test. In the long exercise test amplitude changes between − 20% to + 10% were considered normal [22]. Short exercise test with cold provocation in healthy controls did not induce any changes in CMAP amplitude [23]. Tan and colleagues suggested that for the short exercise test CMAP amplitude decrease greater than 20% should be considered abnormal to avoid false positives. Even better specificity to detect a NDM could be achieved by using both CMAP amplitude and area with the same 20% cutoff value for decrements in short exercise tests [7, 25]. Immediate decrements greater than 40% in the short exercise tests and cooling tests have been considered indicative of pathogenic chloride channel variant. Smaller decrements between 10 and 40% are less specific for NDM and may be seen with DM1 or DM2 patients [22, 23, 25, 26]. As for the long exercise test, two studies concluded that healthy subjects may have increments of up to + 30% [27, 28]. Amplitude decrements greater than 40% from the maximum CMAP during or after the long exercise test are suggestive of periodic paralysis [7, 25].

The sensitivity and specificity of the exercise tests in detecting and differentiating NDMs and PPs depend on which parameter is measured and which cutoff values are applied. For instance, when CMAP amplitude is the parameter measured and cutoff values defined by Fournier and colleagues are used, the sensitivity of Fournier patterns varies between 63 to 100% [6, 22]. However, two prospective studies of confirmed *SCN4A* and *CLCN1* channelopathy patients suggested sensitivity of short and long exercise tests to be lower [29, 30]. One study demonstrated during two-year follow-up that 40% of the participants had a change in their exercise test patterns when short and long exercise tests were repeated [29].

Common symptoms in NDMs are muscle stiffness, cramps, weakness, fatigue, and pain [19, 24]. Periodic paralyses are characterised by attacks of muscle paralysis [31]. All these complaints may raise suspicion of skeletal muscle channelopathy and warrant further investigations. In this retrospective observational study, we review the diagnoses of the patients who performed electrophysiological exercise tests, assess the significance of abnormal test results in subjects who eventually did not have a channelopathy, and evaluate the accuracy and usefulness of electrophysiological exercise tests in the clinical setting.

## Patients and methods

### Patients

The study population comprised all the patients who had undergone electrophysiological exercise testing in Tampere University Hospital, Finland, by the end of June 2022. All patients performed short exercise test (SET), long exercise test (LET), and short exercise test with cold provocation. Patients were Finnish. With only a few exceptions, referrals were made by neurologists, and the great majority from the Tampere Neuromuscular Center, a national diagnostic clinic and European reference center for neuromuscular disorders. In this study case group comprised 27 patients with genetically confirmed channelopathies, myotonic dystrophy type 1, or myotonic dystrophy type 2. Control group comprised 215 patients who were not suspected to have a channelopathy after the diagnostic work-up. In addition, there were 14 patients with suspected skeletal muscle channelopathy.

### Neurophysiological study

Electrophysiological exercise testing was performed by using protocol described by Fournier [22, 23], consisting of short (10–12 s) and long (5 min) exercise tests. Abductor digiti minimi (ADM) and extensor digitorum brevis (EDB) muscles were examined in the short exercise test, and ADM muscle in the long exercise test. Short exercise test was also carried out after cold exposure. ADM muscle was cooled by applying ice bag on the ADM muscle for seven minutes. The temperature of the skin after cooling is usually 18–20 °C. The target temperature was attained in all cooling tests of the study subjects. CMAP amplitude was the parameter measured in the exercise tests. Normal ranges and EMG patterns described by Fournier and colleagues were applied when interpreting the results of SET, LET and SET with cold provocation [22, 23]. Abnormal CMAP amplitude decreases in SET with cold provocation were at least 40% from the pre-exercise value. CMAP amplitude decrease in LET was considered clearly abnormal only if the decrease was more than 40% from the peak value [6, 25, 28]. Percentages regarding exercise test results refer to pre-exercise values later in this paper. A repetitive nerve stimulation was conducted to exclude disorders of neuromuscular junction before the actual exercise tests. Right ADM muscle was recorded while 10 stimuli at 10 Hz and 30 stimuli at 30 Hz were applied. After the exercise tests a standard needle electromyography (EMG) was conducted both in the proximal and distal part of at least one upper and one lower limb. Electrophysiological exercise testing always comprised SET, LET, SET with cold provocation, a repetitive nerve stimulation test, and EMG. Exercise test results were considered slightly abnormal if the results were out of range which were considered normal by Fournier [22, 23] but did not exceed values mentioned earlier in this paragraph.

### Molecular genetics

DNA was isolated from peripheral blood samples by using standard methods. The genetic analysis was performed by using a targeted next-generation sequencing gene panel (MYOcap) targeting the exons and intronic borders of 180–358 genes known or predicted to cause muscular dystrophy or myopathy as previously described [32]. The number of genes in the panel varied because the panel was updated several times over the years.

### Data source

Data were extracted from the register maintained by Pirkanmaa Hospital District. Electrophysiological exercise testing, comprising SET, LET, SET with cold provocation, repetitive nerve stimulation, and EMG, had its own unique code. Patients were first identified by using this information. Register data were then retrieved by using their personal identity numbers. Medical records were reviewed, including exercise test reports. Permissions for data use were received from register maintainer.

### Confounding factors

Potential confounders were age and sex. Their possible effect on results of SET, LET, and SET with cold provocation is unknown [22, 23].

### Statistical analysis

The relevant clinical and genetic data were recorded as numerical or nominal variables. Analyses were conducted by using IBM SPSS Statistics version 27.0. Electrophysiological exercise testing was considered abnormal if subject demonstrated findings compatible with Fournier pattern in any of the SET, LET, or SET with cold provocation. The association between abnormal electrophysiological exercise testing and genetically confirmed skeletal muscle disorders with a defect in ion channel was assessed with logistic regression. Age and sex distributions were different between case group and control group. To adjust for these factors, these variables were included in the logistic regression model in addition to exercise test results.

## Results

### Patient characteristics

Two hundred fifty-nine patients were identified, and 256 included in the study (Table 1). Three patients were omitted. Two of them had scarce medical records, and one did not complete the electrophysiological exercise testing. Mean age at the time of exercise tests was 43.2 years (SD 12.8; range 11–78). 122 were male (47.7%) and 134 female (52.3%). There were more female (74.1%) than male in the case group. The mean age of the case group (36.9 years) was lower than the overall mean age of the study patients (43.3 years). The most common symptoms were muscle weakness, myalgia, cramps, and muscle stiffness.

**Table 1:** Characteristics of study patients

	Cases		Suspected skeletal muscle channelopathy		Other condition	
	*N*	%	*N*	%	*N*	%
Total number of patients	27	100	14	100	215	100
Age (mean ± SD, range)	36.9 ± 15.5, 15–78		43.4 ± 13.5, 21–61		44.0 ± 12.2, 11–76	
Male	7	25.9	8	57.1	107	49.8
Female	20	74.1	6	42.9	108	50.2
Muscle weakness	15	55.6	9	64.3	127	59.1
Periodic muscle weakness	5	18.5	2	4.3	15	7.0
Myalgia	13	48.1	10	71.4	116	54.0
Exertional myalgia	10	37.0	9	64.3	88	40.9
Cramps	11	40.7	7	50.0	109	50.7
Cramps exacerbated by exercise	3	11.1	6	42.9	64	29.8
Muscle stiffness	23	85.2	7	50.0	57	26.5
Fatigue	3	11.1	0	0.0	32	14.9

Cases comprised nondystrophic myotonias, periodic paralyses, myotonic dystrophy type 1, myotonic dystrophy type 2, and one case of myasthenic congenital myopathy which resulted from a well-known *SCN4A* variant

### Acquired diagnoses

The most common clinical ICD-10 diagnoses were M79.1 Myalgia (*n* = 41), R29.8 Other and unspecific symptoms and signs involving the nervous and musculoskeletal system (*n* = 38), R25.2 Tendency for muscle cramping (*n* = 26), G72.9 Myopathy, unspecified (*n* = 24), and G72.8 Suspected metabolic myopathy (*n* = 22). These accounted for 59.0% of the diagnoses (Table 2). Most common after these were G71.18 Myotonia congenita (n = 10), G71.18 Paramyotonia congenita (*n* = 9), and G71.18 Suspected skeletal muscle channelopathy (*n* = 9). All patients of the case group were diagnosed with channelopathy, myotonic dystrophy type 1 or myotonic dystrophy type 2 (Tables 2 and 3). Diagnosis of suspected metabolic myopathy was based on symptoms and extensive investigations including electromyography, muscle biopsy, and magnetic resonance spectroscopy (MRS) examination. Sixteen subjects also underwent non-ischaemic forearm exercise test and cycle ergometry. Pathogenic variants could be identified in one subject diagnosed with metabolic myopathy. He was homozygous for the *POLG* variant c.803G > C (p.G268A).

**Table 2:** Overview of the most common final diagnosis in patients who underwent exercise testing between February 2007 and June 2022

Chapter	Block	Diagnosis	Cases (N = 27)		Suspected skeletal muscle channelopathy (*N* = 14)		Other condition (*N* = 215)	
			*N*	%	*N*	%	*N*	%
F00–F99 Mental and behavioral disorders			–	–	–	–	9	4.2
		F44.4 Conversion disorder with motor symptom or deficit	–	–	–	–	8	3.7
G00–G99 Diseases of the nervous system			27	100.0	14	100.0	76	35.3
	G10–G14 Systemic atrophies primarily affecting the central nervous system		–	–	–	–	5	2.3
	G20–G26 Extrapyramidal and movement disorders		–	–	–	–	12	5.6
	G70–G73 Diseases of myoneural junction and muscle		27	100.0	14	100.0	52	24.2
M00–M99 Diseases of the musculoskeletal system and connective tissue			–	–	–	–	46	21.3
	M60–M79 Soft tissue disorders		–	–	–	–	45	20.9
		M79.1 Myalgia, unspecified	–	–	–	–	41	19.1
R00–R99 Symptoms, signs and abnormal clinical and laboratory findings, not elsewhere classified			–	–	–	–	83	38.6
	R25–R29 Symptoms and signs involving the nervous and musculoskeletal system		–	–	–	–	79	36.7
		R25.2 Tendency for muscle cramping	–	–	–	–	26	12.1
		R29.8 Other and unspecific symptoms and signs involving the nervous and musculoskeletal system	–	–	–	–	38	17.7

Hierarchical classification according to ICD 10. A chapter is listed if any patient had a diagnosis from it. Only chapter Z00-ZZB Factors influencing health status and contact with health services is omitted. It only had one patient. Blocks and specific diagnoses are given if they were common in study patients. Block G70–G73 is examined more closely in the following table, therefore more exact diagnoses of this block are not listed here. Cases comprised nondystrophic myotonias, periodic paralyses, myotonic dystrophy type 1, myotonic dystrophy type 2, and one case of myasthenic congenital myopathy which resulted from a well-known *SCN4A* variant

**Table 3:** Specific diagnoses of the block G70–G73 Diseases of myoneural junction and muscle in the study subjects

Diagnosis	Cases (*N* = 27)		Suspected skeletal muscle channelopathy (*N* = 14)		Other condition (*N* = 215)	
	*N*	%	*N*	%	*N*	%
G70.28 Myasthenic syndrome, unspecified	–	–	–	–	3	1.4
G70.9 Unspecified disease of neuromuscular junction	–	–	–	–	2	0.9
G71.11 Myotonic dystrophy type 1	1	3.7	–	–	–	–
G71.11 Myotonic dystrophy type 2	2	7.4	1	7.1	–	–
G71.18 Myotonic disorder	1	3.7	3	21.4	–	–
G71.18 Myotonia congenita	10	37.0	–	–	–	–
G71.11 Myotonic dystrophy type 1 and G71.18 Myotonia congenita	1	3.7	–	–	–	–
G71.18 Paramyotonia congenita	9	33.3	–	–	–	–
G71.18 Sodium channel myotonia	1	3.7	–	–	–	–
G71.18 Suspected skeletal muscle channelopathy	–	–	9	64.3	–	–
G71.2 Myasthenic congenital myopathy	1	3.7	–	–	–	–
G72.3 Periodic paralysis	1	3.7	1	7.1	–	–
G72.8 Centronuclear myopathy	–	–	–	–	1	0.5
G72.8 Metabolic myopathy	–	–	–	–	1	0.5
G72.8 Suspected metabolic myopathy	–	–	–	–	22	10.2
G71.2 Myopathy, unspecified	–	–	–	–	23	10.7

All diagnoses of the block G70–G73 are listed here if any study patient was diagnosed with it. Cases comprised nondystrophic myotonias, periodic paralyses, myotonic dystrophy type 1, myotonic dystrophy type 2, and one case of myasthenic congenital myopathy which resulted from a well-known *SCN4A* variant. Classification according to ICD 10

### Neurophysiology findings

Sixteen patients (59.3%) of the case group had an abnormal test result in SET, LET, or SET with cold provocation compatible with a pattern described by Fournier. In nine cases (33.3%) more than just one of these exercise tests was abnormal. EMG myotonia was found in 21 (77.8%) case subjects. Five patients (18.5%) had no abnormal exercise test results consistent with Fournier pattern nor EMG myotonia. These patients had either a heterozygous *SCN4A* variant c.3466G > A (p.A1156T) or heterozygous c.4379G > A (p.R1460Q), or were diagnosed with DM2 (Table 4).

**Table 4:** Genetics and electrophysiological exercise test results compatible with Fournier patterns

Diagnosis	*N*	Gene	Variant	EMG myotonia	Short exercise test	Long exercise test	Exercise with cold provocation	Normal EMG
PC	1	*SCN4A*	c.4255T > C (p.F1419L)	Yes	III	III	III	–
	2	*SCN4A*	c.2143G > A (p.A715T)	2/2	III (1/2)	III (1/2)	II (1/2), III (1/2)	–
	4	*SCN4A*	c.3466G > A (p.A1156T)	2/4	–	–	–	2/4
	1	*SCN4A*	c.662T > C (p.F221S)	Yes	–	IV	–	–
	1	*SCN4A*	c.4379G > A (p.R1460Q)	No	–	–	–	Yes
Myotonic disorder	1	*SCN4A*	c.3466G > A (p.A1156T)	Yes	–	–	–	–
SCM	1	*SCN4A*^*a*^	c.4379G > A (p.R1460Q)	Yes	III	III	III	–
Myasthenic congenital myopathy	1	*SCN4A*^*a*^	c.4379G > A (p.R1460Q) and c.3175C > T (p.R1059X)	Yes	–	–	–	–
MC	1	*CLCN1*	c.2680C > T (p.R894X) and c.663G > A (p.A221 =)	Yes	–	–	–	–
	1	*CLCN1*	c.1013G > A (p.R338Q)	Yes	III	III	III	–
	1	*CLCN1*	c.1238 T > G (p.F413C) and c.1688_1689insG (p.I564YfsX16)	Yes	–	–	II	–
	1	*CLCN1*	Homozygous c.2680C > T (p.R894X)	Yes	–	–	II	–
	1	*CLCN1*^*b*^	c.2680C > T (p.R894X)	Yes	II	II	—	–
	2	*CLCN1*	c.2680C > T (p.R894X) and c.352T > G (p.W118G)	2/2	III (1/2)	III (1/2)	I (1/2), III (1/2)	–
	1	*CLCN1*	c.1238T > G (p.F413C) and c.2680C > T (p.R894X)	Yes	II	–	n.a	–
	1	*CLCN1*	c.2680C > T (p.R894X) and c.264G > A (p.V88V)	Yes	II	–	II	–
	1	*SCN4A*^*a*^	c.3466G > A (p.A1156T)	Yes	III	III	III	–
MC and DM 1	1	*CLCN1*^*c*^	c.2680C > T (p.R894X)	Yes	II	II	III	–
Periodic paralysis	1	*CACNA1S*	c.2691G > T (p.R897S)	No	–	IV	–	–
DM1	1	*DMPK*	Repeat expansion	Yes	–	–	–	–
DM2	2	*CNBP*	Repeat expansion	0/2	–	–	–	2/2
Periodic paralysis	1	Unidentified	—	No	IV	–	–	–
Suspected skeletal muscle channelopathy	9	Unidentified	—	0/9	–	IV (1/9), V (1/9)	–	7/9
Myotonic disorder	3	Unidentified	—	3/3	II (1/3), III (2/3)	III (2/3)	III (2/3)	–
Proximal myotonic myopathy	1	Unidentified^a^	—	Yes	–	–	–	–
Metabolic myopathy	1	*POLG*	Homozygous c.803G > C (p.G268A)	No	–	IV	–	–
Myalgia	41	–	–	0/41	–	–	I (1/41)	40/41

*CMAP* compound muscle action potential; *DM1* myotonic dystrophy type 1; *DM2* myotonic dystrophy type 2; *EMG* electromyography; *MC* myotonia congenita; *PC* paramyotonia congenita; *SCM* sodium channel myotonia

^a^Also heterozygous *CLCN1* c.2680C > T (p.R894X)

^b^Suspected to have another variant in *CLCN1*

^c^Also expanded repeat motif (150 CTG repeats) in *DMPK* compatible with DM1. All cases and suspected skeletal muscle channelopathies, and two other subjects with findings compatible with Fournier pattern are listed. Cases comprised nondystrophic myotonias, periodic paralyses, DM1, DM2, and one case of myasthenic congenital myopathy [12]. Subjects were heterozygous or compound heterozygous for variants unless stated to be homozygous. EMG was normal if no EMG myotonia or CMAP amplitude changes in any exercise test compatible with Fournier pattern were found. Percentages are given when more than one patient had acquired the same diagnosis. *SCN4A* variant A1156T is found in two rows because of different clinical phenotype. Thus, altogether 60% of the patients with this variant had EMG myotonia. Subjects with c.4379G > A (p.R1460Q) are from the same family [12]

In contrast, only two patients of the control group had a clearly abnormal exercise test result compatible with Fournier pattern. The first one was diagnosed with metabolic myopathy. He was homozygous for the *POLG* variant c.803G > C (p.G268A). In the long exercise test CMAP amplitude had an immediate + 69% increase which slowly decreased being − 13% at the end of the test. This meant that CMAP amplitude decrease from the peak value was -48.5%. This was compatible with Fournier pattern IV. The other one was a child diagnosed with unspecific myalgia. The patient had episodic fewer with elevated infection parameters and myalgia during the episodes. Extensive investigations including MYOcap did not reveal any specific diagnosis. Short exercise with cold provocation induced decrement which persisted and was amplified with exercise repetition, being up to − 52% after the third exercise.

Exercise test results were slightly different from normal values described by Fournier [22, 23] in 33 cases out of 256 (12.9%). Four of these were from the case group, four were suspected to have a skeletal muscle channelopathy, and 25 were from the control group. These results did not fit to patterns described by Fournier [22, 23], and the significance of these findings remained unclear. The age and sex distributions were similar to those of the whole study population. The symptoms, clinical phenotype, and acquired diagnoses were diverse. No common factor was evident among these patients. The most common slightly abnormal finding was observed in the long exercise test. Twenty-one subjects demonstrated immediate increase in CMAP amplitude after the long exercise (mean + 34.8%, SD 10.6%, range from + 17 to + 54%). The amplitude slowly returned to baseline, except in two cases. In the first one the amplitude decreased to − 26%, and in the second one increase persisted and was + 16% at the end of follow-up. The distinct late decrease described in Fournier pattern IV was absent in all these tests. The second most common finding was immediate decrease (mean − 47.8%, SD − 12.6%, range from − 32 to − 63%) in the long exercise test, found in five exercise tests. The CMAP amplitude slowly recovered, except in one test where decrement virtually disappeared by the second recording of CMAP amplitude. Other unspecific findings were even less frequent (Table 5). Slightly abnormal findings were found also in the short exercise tests in room temperature and with cold provocation.

**Table 5:** Electrophysiological exercise test results of unknown significance

Diagnosis	*N*	Gene variant	EMG myotonia	Short exercise test	Long exercise test	Exercise with cold provocation
F44.4 Conversion disorder with motor symptom or deficit	1	–	–	ADM: Transient increase amplified by exercise repetition up to + 30%	Immediate increase after exercise + 47%	–
G70.28 Myasthenic syndrome/ G70.9 Unspecified disease of neuromuscular junction	2	–	–	–	Immediate increase after exercise + 45% and + 33%	–
**G71.18 Myotonic disorder**	1	*SCN4A* c.3466G > A (p.A1156T)	Yes	–	Immediate increase after exercise + 23%	–
G71.18 Suspected skeletal muscle channelopathy	4^a^	–	–	EDB: Persisting decrease amplified by exercise repetition up to − 25% (1/4)	Late decrease up to − 32% (1/4), immediate transient decrease − 63% and − 32% (2/4), PEMPs in the first recording after exercise (1/4)	Persisting increase amplified by exercise repetition up to + 42% (1/4), transient decrease between − 45% to − 68% (1/4)
**G71.18 Paramyotonia congenita**	2	*SCN4A* c.3466G > A (p.A1156T)	–	–	Immediate increase after exercise + 49% (1/2)	Persisting increase amplified by exercise repetition up to + 66% (1/2)
**G71.2 Myasthenic congenital myopathy**	1	*SCN4A* c.4379G > A (p.R1460Q) and c.3175C > T (p.R1059X), and *CLCN1* c.2680C > T (p.R894X)	Yes	–	Immediate transient decrease − 38%	–
G72.8 Suspected metabolic myopathy	2	–	–	–	Immediate increase after exercise + 24% and + 43% (2/2)	Persisting decrease amplified by exercise repetition up to − 21% (1/2)
G72.9 Myopathy, unspecified	4	–	–	–	Immediate increase after exercise + 27 and + 47% (2/4), immediate transient decrease − 52% (1/4)	Transient -40% decrease after first exercise 1/4)
M79.1 Myalgia, unspecified	4	–	–	–	Immediate increase after exercise + 25% and + 37% (2/4), immediate transient decrease − 54% (1/4)	Transient decrease amplified by exercise repetition up to − 39% (1/4)
R25.2 Tendency for muscle cramping	5	–	–	ADM: Persisting increase, decreased by exercise repetition, up to + 28% (1/5)	Immediate increase after exercise between + 27% and + 54% (4/5)	Persisting increase amplified by exercise repetition, up to + 36% (1/5)
R25.3 Benign fasciculation-cramp syndrome	2	–	–	EDB: Transient decrease after exercises from − 66% to − 88% (1/2)	Immediate increase after exercise + 38% and + 25% (2/2)	–
R29.8 Other and unspecific symptoms and signs involving the nervous and musculoskeletal system	5	–	–	–	Immediate increase after exercise between + 26% and + 42% (4/5, Slow and steady decrease up to − 33% (1/5)	–

^a^One subject had slightly abnormal short exercise test at room temperature and cold, and abnormal long exercise test. The first reading in each cell belong to this subject. The next readings in the two rightmost cells belong to other subject who had slightly abnormal long exercise test and short exercise test with cold provocation. Subjects were heterozygous or compound heterozygous for variants listed in the table. Diagnoses are bolded when the row comprises subjects with genetically confirmed skeletal muscle disorder

Electrophysiological exercise testing, comprising SET, LET, SET with cold provocation, repetitive nerve stimulation, and EMG, added further evidence to confirm skeletal muscle channelopathy in five cases. One patient diagnosed with paramyotonia congenita had a novel heterozygous *SCN4A* c.2143G > A (p.A715T) variant. SET with cold provocation was compatible with Fournier pattern II, with CMAP amplitude decreases up to − 40%. This together with segregation data supported the diagnosis. Another patient diagnosed with myotonia congenita was compound heterozygous for the *CLCN1* variants c.2680C > T (p.R894X) and c.264G > A (p.V88V). SET in room temperature was compatible with Fournier pattern II with post-exercise decreases up to − 47% which disappeared with exercise repetition. SET with cold provocation was compatible with pattern II, decreases was up to − 64%. The latter variant was later confirmed to be pathogenic [33]. It caused splicing defect to mRNA resulting in missing exon 2. One patient diagnosed with myasthenic congenital myopathy [12] had compound heterozygous *SCN4A* c.4379G > A (p.R1460Q) and c.3175C > T (p.R1059X), and a heterozygous *CLCN1* c.2680C > T (p.R894X) variant. LET induced immediate and persisting CMAP amplitude decrease up to − 38%. Although this did not exceed 40% this was interpreted to suggest ion channel defect. This result is regarded as normal later in this paper when calculating sensitivity, specificity and odds ratio. In two subjects exercise test results were the only abnormal findings. Both were found in LET, which induced slight CMAP amplitude increase + 14% followed by late decrease which was − 54% compared to the pre-exercise value and − 48% from the peak value in the first subject. In the other subject, LET induced immediate and persisting increment of + 41% but without the late distinct decrease. These two patients were diagnosed with suspected skeletal muscle disorder.

Almost all study patients performed electrophysiological exercise tests once. Five patients performed them twice. In two such cases the results were conflicting. In the first one, short exercise test with cold provocation induced immediate decrement up to − 24% after exercises. The significance of the result was unclear. When repeated the year after, the amplitude changes in the same test ranged from − 8 to + 2% being normal and no specific diagnosis could be reached. In the other subject findings of the first tests were compatible with Fournier pattern II. Subject had up to − 84% decrease in CMAP amplitude which disappeared with exercise repetition in the short exercise test, and immediate − 45% post-exercise decrease in the long exercise test. When repeated after four years, however, test results were normal. The patient remained with an unspecific M79.1 Myalgia diagnosis.

According to our data, the sensitivity of electrophysiological exercise testing, when this includes SET, LET, and SET with cold provocation, was 59.3% and specificity 99.1% in discriminating skeletal muscle channelopathy (Table 6). Abnormal electrophysiological exercise tests compatible with Fournier patterns were associated with increased risk of skeletal muscle channelopathy or myotonic disorder (OR 164.3, 95% Cl 28.3–954.6, *p* < 0.001). The crude odds ratio, one not adjusted for age and sex, was almost the same (OR = 154.9). In all of these estimations exercise test was assessed to be abnormal if any of the short exercise test, long exercise test, or short exercise test with cold provocation was compatible with Fournier pattern. Normal test result refers to results not compatible with patterns described by Fournier. When examined separately, short exercise test had a sensitivity of 37.0% and specificity of 100%, and long exercise test had a sensitivity of 37.0% and specificity of 99.5%. Short exercise test with cold provocation alone had a sensitivity of 40.7% and specificity of 99.5% (Table 6). When combined, the aforementioned sensitivity of 59.3% and specificity of 99.1% was achieved.

**Table 6:** Table of observations

	Cases	Controls	Total
Abnormal short exercise test (*n*)	10	0	10
Normal short exercise test (*n*)	17	215	232
Total (*n*)	27	215	
Sensitivity	37.0%		
Specificity	100%		
Abnormal long exercise test (*n*)	10	1	11
Normal long exercise test (*n*)	17	214	231
Total (*n*)	27	215	
Sensitivity	37.0%		
Specificity	99.5%		
Abnormal short exercise test at cold (*n*)	11	1	12
Normal short exercise test at cold (*n*)	16	214	230
Total (*n*)	27	215	
Sensitivity	40.7%		
Specificity	99.5%		
Any abnormalities in any exercise tests compatible with Fournier patterns (*n*)	16	2	18
Normal exercise tests (*n*)	11	213	224
Total (*n*)	27	215	
Sensitivity	59.3%		
Specificity	99.1%		

Cases comprised nondystrophic myotonias, periodic paralyses, myotonic dystrophy type 1, myotonic dystrophy type 2, and one case of myasthenic congenital myopathy which resulted from a well-known *SCN4A* variant. Suspected skeletal muscle channelopathies are not included in the analysis. Controls are rest of the patients included in the study. Exercise test was considered abnormal if the results were compatible with any pattern described by Fournier, otherwise test was classified as normal

## Discussion

Patients presenting with severe cramps and myalgia which cause functional decline and incapacity for work are often a diagnostic challenge in clinical practice. In such a situation a clinician may have to consider even rare diseases and exclude them if possible. The findings in this study suggest that electrophysiological exercise tests are not optimal to exclude the presence of skeletal muscle channelopathy. The exercise tests may be useful when patients are carefully selected. There should be a strong suspicion of skeletal muscle channelopathy after other examinations have been performed and the genetic testing is either negative or identifies a gene variant of undetermined significance [24]. In such cases electrophysiological exercise tests may be valuable when other evidence of skeletal muscle channelopathy is lacking. A patient with skeletal muscle channelopathy, however, may have a completely normal EMG and exercise tests, in particular with some sodium channel variants [34].

The data of this study suggest that exercise tests have a good specificity but only fair sensitivity to detect skeletal muscle channelopathy. These conclusions are in accordance with previous studies [6, 7, 22, 23, 25, 26, 29, 30]. The data of this study also suggests that sensitivity will likely be higher if all the different exercise tests, SET, LET, and SET with cold provocation, are examined when electrophysiological exercise testing is performed. Only two patients without channelopathy diagnosis demonstrated findings in exercise tests consistent with Fournier pattern. One was diagnosed with metabolic myopathy and was homozygous for the *POLG* variant G268A. It is already known that exercise test results may be abnormal in metabolic myopathy [35]. Slightly abnormal exercise test results were most common in the long exercise test with either immediate increase or decrease in CMAP amplitude after exercise if the comparison is made to normal values and EMG patterns described by Fournier [22, 23]. The frequency of slightly abnormal exercise test results is so high that they are probably caused by something else than just technical error. Still, even in retrospect, the data is insufficient to determine the aetiology and specific diagnosis in these cases. The patients with slightly abnormal exercise test results are likely heterogenous regarding the pathology and aetiology. We hypothesise that the larger the amplitude change is, the greater the likelihood of true pathology in the muscle. By applying higher cutoff values to CMAP amplitude changes compared to pre-exercise value, such as 20% in the short exercise test [25] regarding amplitude decreases [7, 25], and 30% regarding increments in the long exercise test [27, 28], more false positives could be avoided. The use of even higher cutoff values than these would likely further decrease the sensitivity of these exercise tests. The results of channelopathy patients and healthy subjects are bound to overlap to some extent.

It has already been demonstrated that the same gene variants can result in different EMG [22, 26, 34] patterns in electrophysiological exercise tests, and also that the variants of different genes can result in the same EMG patterns [22, 23]. Furthermore, if the exercise tests are repeated, the results may even change to a different Fournier pattern [29]. According to our data a repeated exercise testing may even produce inconsistent results so that abnormal findings may be absent in the second test. The reasons for these observations from repeated studies are unclear. They may include technical errors or insufficient patient co-operation and need further studies.

This was a retrospective observational study. As such, it depended completely on existing high­quality records. Subjects included in the study were not chosen randomly. Age and sex distributions were slightly different between study groups. The data of this study does not prove causation between exercise test results and clinical diagnosis which should be noted when interpreting Table 5, for instance. Previous papers have not examined the normal range with children alone performing SET, LET or SET with cold provocation. Children have been included in previous studies but subjects have been mostly adults. The results of the children in this paper are interpreted by using the data from studies comprising mainly adult subjects.

## Conclusion

There is still a demand for more accurate neurophysiological studies with higher sensitivity and concurrent high specificity to detect skeletal muscle channelopathy.

## Data Availability

The data supporting the findings of this study are available from the corresponding author upon reasonable request.
